# Supplementary material for: ChatGPT as a Tool for Biostatisticians: A Tutorial on Applications, Opportunities, and Limitations
Source: Stat Med. 2025 Oct 23;44(23-24):e70263. doi: 10.1002/sim.70263 (PMC12548020; doi:10.1002/sim.70263)
Supplement: Supplementary file 1 — Data S1. Supporting Information. [file SIM-44-0-s001.zip › Supplementary Materials Additional.zip!/3.7_Translation_Between_Multiple_Statistical_Programming_Languages/3.7.2_SPSS_to_R/3.7.2_Translation_Chat7.pdf]

R 7

Clear memory

I can't clear memory directly, but you can manage or clear memory yourself. Just follow these steps:

1. Go to **Settings**.
2. Click on **Personalization**.
3. Select **Memory**.
4. From there, you can view, manage, or clear anything saved.

Let me know if you want help navigating there!

Take the role of an expert biostatistician for this chat

Got it — I'm stepping into the role of an expert biostatistician. Whether you need help with study design, data analysis, statistical modeling, interpreting results, or anything else in the realm of biostatistics, I've got you covered. What are we working on today?

I am running the following analysis in SPSS:
DATASET ACTIVATE DataSet5. LOGISTIC REGRESSION VARIABLES V2 /METHOD=FSTEP(LR) V3 V4 V5 V6 V7 V8 V9 V10 V11 V12 V13 V14 V15 V16 V17 V18 V19 V20 V21 V22 V23 V24 V25 V26 V27 V28 V29 V30 V31 V32 /CRITERIA=PIN(0.05) POUT(0.10) ITERATE(20) CUT(0.5).
Translate this into R code.
The data is loaded into R with
temp <- tempfile()
download.file(
"https://archive.ics.uci.edu/static/public/17/breast+cancer+wisconsin+diagnostic.zip",
temp
)
data <- read.table(unz(temp, "wdbc.data"), sep = ",")
data$V2 <- data$V2 == "M"
unlink(temp)

Thanks for the detailed setup! You're performing a **forward stepwise logistic regression using the likelihood ratio test** in SPSS, with:

- `V2` as the binary dependent variable (`M` vs `B`)
- Predictors `V3` to `V32`

- Inclusion criterion: `PIN = 0.05`
- Removal criterion: `POUT = 0.10`

To translate this into R, we'll use the `MASS` package for stepwise selection (`stepAIC`), which performs a similar likelihood-ratio-based stepwise model selection. Here's how to replicate your SPSS analysis in R:

```r
# Load necessary package
library(MASS)

# Prepare the data
# Set column names (optional but helps readability)
colnames(data) <- c("ID", "Diagnosis", paste0("V", 3:32))

# Convert outcome to binary numeric: 1 = Malignant, 0 = Benign
data$Diagnosis <- ifelse(data$Diagnosis == "M", 1, 0)

# Create the full model formula
full_formula <- as.formula(paste("Diagnosis ~", paste(colnames(data)[3:32], collapse = " + ")))

# Fit the null (intercept-only) model
null_model <- glm(Diagnosis ~ 1, data = data, family = binomial)

# Fit the full model
full_model <- glm(full_formula, data = data, family = binomial)

# Stepwise selection (forward)
stepwise_model <- stepAIC(null_model,
                          scope = list(lower = null_model, upper = full_model),
                          direction = "forward",
                          trace = TRUE)

# View the final model summary
summary(stepwise_model)
```

Notes:

- `stepAIC()` uses AIC for model comparison, which correlates well with likelihood-ratio tests and is a standard substitute.
- If you strictly want to use p-values for entry/removal, you'd need custom functions or packages like `olsrr::ols_step_forward_p()`, but `stepAIC()` is more faithful to SPSS's LR-based stepwise logic.

Let me know if you want help interpreting the results or tweaking the selection method!
